# Levels of Adherence of an Exercise Referral Scheme in Primary Health Care: Effects on Clinical and Anthropometric Variables and Depressive Symptoms of Hypertensive Patients

**DOI:** 10.3389/fphys.2021.712135

**Published:** 2021-12-21

**Authors:** Katia Gallegos-Carrillo, Carmen Garcia-Peña, Nelly Salgado-de-Snyder, Jorge Salmerón, Felipe Lobelo

**Affiliations:** ^1^Unidad de Investigación en Epidemiología y Servicios de Salud, Instituto Mexicano del Seguro Social (IMSS), Cuernavaca, Mexico; ^2^Instituto Nacional de Geriatría, Mexico City, Mexico; ^3^Centro de Investigación en Sistemas de Salud, Instituto Nacional de Salud Pública (México), Cuernavaca, Mexico; ^4^Academic Unit of Epidemiological Research, National Autonomous University of Mexico, Mexico City, Mexico; ^5^Rollins School of Public Health, Emory University, Atlanta, GA, United States

**Keywords:** health behaviors, physical activity, hypertension, adherence, primary care (MeSH)

## Abstract

Among the modifiable health behaviors, physical activity (PA) promotion has been one of the challenges in primary care, particularly how to translate the results of proven interventions and implement them in the real world. This study was aimed to compare whether two programs designed for hypertensive patients achieve changes in clinical and anthropometric variables, quality of life, and depressive symptoms; and if higher levels of adherence to one of the interventions using an exercise referral (ER) approach achieved better health outcomes. Pragmatic cluster randomized trials were carried out in four Primary Health Care Units (PHCUs). Physicians in the PHCUs identified hypertensive patients and assessed whether they were eligible to be part of this trial. Each center was randomized to a brief PA counseling (BC, *n* = 2) or an exercise referral (ER, *n* = 2) intervention to conducted PA programs among hypertensive patients aged 35–70 years, self-reported as physically inactive. Outcome variables included changes in blood pressure levels, triglycerides, HDL cholesterol, fasting glucose, body mass index, waist/hip ratio, abdominal obesity, and metabolic syndrome risk score, health-related quality of life, and depressive symptoms. Longitudinal multilevel analyses assessed the effects of the BC and ER programs and the level of adherence of the ER on clinical, anthropometric, and mental health variables, models were linear for continuous variables, and logistic for dichotomous variables. Differences were observed in triglycerides, BMI, metabolic risk scores variables, and depressive symptoms among ER and BC programs. In addition, differences in the ER group were observed according to the level of adherence in blood pressure levels, waist circumference and waist/hip ratio, depressive symptoms, and the mental health component of health-related quality of life. An ER program in comparison to a BC intervention is promoting changes in some specific health indicators of hypertensive patients, showing the usefulness of these PA programs in primary health care facilities.

## Introduction

In the global health community, one of the greatest challenges is how to translate the results of proven interventions and implement them in the real world. Part of this challenge resides on the individuals themselves who are responsible for making these recommendations.

The efficacy of interventions to reduce high-risk behaviors through healthy lifestyle promotion, including healthy food habits, smoking cessation, and increased physical activity (PA) levels, has been determined in randomized or quasi-randomized controlled trials (Ashenden et al., 1997; Lawlor and Hanratty, 2001; Eden et al., 2002; Stead et al., 2016). However, some studies reveal that interventions aimed to change unhealthy health behaviors under realistic conditions of the health service delivery are still challenging to accomplish (Eakin et al., 2004; Aittasalo, 2008).

Among the modifiable health behaviors, the promotion of PA has been one of the challenges in primary care. Estimations indicated that not meeting PA recommendations is responsible for more than 5 million deaths globally each year (Lee et al., 2012). Although regular PA and health levels have a strong causal relationship, only 47% of the general Mexican population is achieving the PA levels. Among the strategies to increase PA levels exercise referral schemes (ERs) provide a promising alternative to PA promotion, facilitating changes in behavior in the at-risk population (Dugdill et al., 2005; Pavey et al., 2011).

Few studies have evaluated the impact of the levels of adherence of PA interventions carried out in primary health care, and its effects on participants physical health, anthropometry, perception of their quality of life, as well as mental well-being. Buckley et al. (2020), considered participant monthly attendance to a fitness center to assess differences of the exercise referral scheme and usual care in the United Kingdom, participants in the usual care program had lower attendance to the program and less attendance sustainability after 6-month follow-up, however, this level of attendance was not used to determine impact of the ER on PA, clinical or anthropometrics outcomes. Other studies in high-income countries have observed moderate effects of the ERS in self-reported PA levels and cardiovascular health outcomes (Murphy et al., 2012; Onerup et al., 2019), while other approaches to assess the effectiveness of ERS through measuring PA adherence concluded that a higher adherence (a more active stage of chance) of those in the ERs compared to usual care show an association with higher levels of PA (Martín-Borrás et al., 2018).

The aim of this study was to compare if two PA programs designed for increasing the PA levels of hypertensive patients achieve changes in clinical and anthropometric variables, quality of life, and depressive symptoms between baseline, 16 and 24 weeks; and if higher levels of adherence to one of the interventions using an exercise referral (ER) approach achieve better outcomes.

## Materials and Methods

### Study Design

Cluster randomized trial, with Primary Health Care Units(PHCUs) as the unit of randomization and hypertensive patients as the unit of assessment.

The physicians in all PHCUs identified hypertensive patients and assessed that they met the established criteria. Study design and details of the recruitment process are described elsewhere (Gallegos-Carrillo et al., 2014).

### Participants

Trained staff in all the PHCUs used the eligibility criteria to warrant both that the practice of PA was safe for all participants, and the conditions to apply the behavioral theory-based intervention: women and men with IMSS affiliation, between 35 and 70 years old, less than 5 years from hypertension diagnosis, and/or without drug treatment (according to JNC 7 criteria) (Chobanian et al., 2003; U.S. Department of Health and Human Services, 2004), self-reported performance of less PA than recommended (<150 min per week of moderate to vigorous intensity PA), and self-reported willingness to practice PA, according to Transtheoretical model of behavioral change (Prochaska and DiClemente, 1983); moderate level of cardiovascular risk, according to the Guidelines for exercise testing and recommendations of the (American College of Sports Medicine, 2009; Thompson et al., 2013), and complemented with a pre-participation screening questionnaire from the American Heart Association (Thomas et al., 1992) and ACSM. Specific information about inclusion criteria has been previously detailed (American College of Sports Medicine, 2009; Thompson et al., 2013; Gallegos-Carrillo et al., 2014).

Hypertensive patients complying with the inclusion criteria were not eligible if they reported a high PA level (>300 min a week of moderate to vigorous intensity), and have previously attended a PA program at IMSS facilities.

### Setting

The Mexican Social Security Institute (IMSS) includes facilities for primary, secondary, and tertiary health care services as well as available resources designed to develop PA programs (such as sports fields, gyms, pools, and outdoor spaces for PA) called Social Security Centers (SCC). The screening assessment and the exercise reference by physicians were carried out in PHCUs, while the PA program exercise program was performed during 16 weeks at SCCs. The functioning and staff of the SCCs were described previously.

### Sample Size

The sample size estimation was based on the goal of increasing the percentage of hypertensive patients who are physically active from 17 to 37%, the proportion that has been shown in preliminary assessments. An increase in compliance with PA recommendations of 20% showed whether the intervention was effective. The proportion is consistent with the effectiveness level tested in other studies (Harrison et al., 2005). Thus, to identify a difference of 20% more of the population becoming physically active, with a power of 90, and a level of significance of α = 0.05, 68 patients were required in each group (IG and CG). We considered increasing our sample size by 40%, due to the high dropout rates reported in previous studies (Hillsdon et al., 2004). Therefore, the sample size was estimated to be 224 hypertensive patients, 112 for each group. We assumed that a PA program with planned sessions of PA and surveillance as it is the Exercise Referral Scheme (ER), would be a better approach to achieve better health outcomes at the end of the intervention than just brief counseling (BC).

### Randomization

To avoid contamination randomization was conducted with IMSS facilities PHCUs located on average 5.5 kilometers (kms) from the place where patients were referred (ER at 5.1 kms, and BC at 6.6 kms), within the urban area of the city, and had public transportation available, average transfer time being 20 to 30 min. The four PHCUs were randomized into an intervention group called exercise referral (ER) (two centers) and a BC (two centers) using sealed envelopes by a health researcher who was not involved in this study.

### Recruitment Process

**FIGURE 1:**
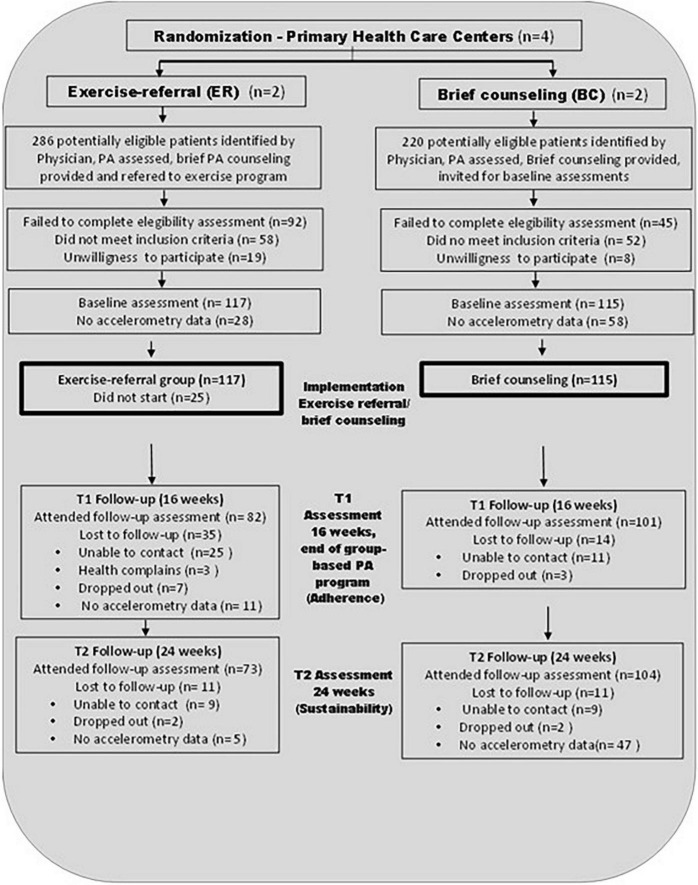
Consort flow diagram.

The recruitment process took place from September 2011 to March 2012, in four PHCUs; 506 hypertensive patients were identified and assessed by 108 PHC physicians who took part in the four PHCUs that were randomized as the study groups. The activities of the physicians during the daily consultation to identify potential participants were expressed in a previous publication (Gallegos-Carrillo et al., 2014). Figure 1 shows the flow diagram detailing the recruitment of participants during the study, according to guidelines of the Consolidated Standard of Reporting Trials (CONSORT) (Schulz et al., 2010). During recruitment, we identified 506 hypertensive patients as potential candidates for the study for both groups, ER and BC; of them, 21.7% were excluded because their levels of blood pressure were >160 mmHg systolic, or >100 mmHg diastolic during screening. In addition, 5.3% declined to participate, and 27.1% were unable to attend or complete assessments. Therefore, at the end of the recruitment process, 117 patients (50.4 years old on average and 67.5 % women) were incorporated into the intervention group and 115 into the control group (51.7 years old on average and 73.04 % women).

### Outcome Measures

The main health outcomes were: systolic and diastolic blood pressure levels, triglycerides, HDL cholesterol, fasting glucose, body mass index, weight, waist/hip ratio, and metabolic syndrome risk score, health-related quality of life, and depressive symptoms.

Follow-up measurements were carried out at the end of the intervention (16 weeks) for both ER and BC groups, while the sustainability of intervention effects was assessed 24 weeks after the beginning of the study. In addition, differences in health outcomes were observed among hypertensive patients belonging to the ER group who achieved 50% of adherence to the PA program, which means they attended at least 24 planned PA sessions or more.

(a) Blood pressure levels measured with a digital instrument following criteria established by the JNC 7 (Chobanian et al., 2003; U.S. Department of Health and Human Services, 2004);
(b) Biochemical markers measured according to the procedures of the International Federation of Clinical Chemistry and Laboratory Medicine (Tate et al., 1999): (1) Fasting glucose levels: blood samples were taken following a fasting period of at least 8 h. The serum glucose determination was made using the enzymatic calorimetric method. All blood biochemistry tests, including (2) total cholesterol, (3) high-density lipids (HDL), and (4) triglycerides were conducted with a Selectra XL (Randox).
(c) Anthropometric measures were carried out by trained nurses, following standard procedures: (1) weight, measured with TANITA electronic scale, with participants wearing minimal clothes and without shoes; (2) Body Mass Index (BMI), was calculated dividing kilograms by height in meters squared. The data obtained were categorized according to the following criteria: normal (BMI = 18.5–24.9 kg/m^2^), overweight (BMI = 25–29.9 kg/m^2^) and obese (BMI = > 30 kg/m^2^) (World Health Organization [WHO], 1995); (3) waist circumference, measured at the highest point of the iliac crest at the end of expiration, to the nearest measuring tape point of 0.1 cm. The criteria for abdominal obesity will be: men >100 cm and women >88 cm (Expert panel, 2001); and (4) hip circumference, participants were stood with feet separated about 20 cm and weight distributed evenly on both feet, at the level of the maximum extent of the gluteus in a horizontal plane, verifies that the measuring tape covered at same high the perimeter of the body, near to the skin but without compress (World Health Organization [WHO], 2000) and waist/hip ratio.
(d) Physical and psychological measurements: (1) health-related quality of life (HRQoL), measured through the short form of 12 items (SF-12), considering two components physical (PCS) and mental (MCS) (Ware et al., 1996). The Physical Activity Summary Measures (PCS) included four scales, whose scores varied from 0 to 100, which were summarized to generate the PCS, a higher score meaning better PA functioning and fewer limitations. The scales are the following: Physical Functioning (PF), Role-Physical (RP), Bodily Pain (BP), and General Health (GH). While the Mental Health Activity Summary Measure (MCS) included four scales which also varied between 0 and 100 in which a higher score represents better mental health functioning: Vitality (VT), Social Functioning (SF), Role-Emotional (RE), and Mental Health (ME) were the criteria.

(2) Symptoms of depression were measured with the Center for Epidemiologic Studies–Depression Scale, (CESD), this version included 20 items which are summarized to create the scale, the score range is from 0 to 60, higher scores are indicating a certain level of depressive symptomatology (Radloff, 1977).

Metabolic Syndrome Risk Score (MetSyn): Abdominal obesity; elevated fasting glucose as 100 mg/dL, triglycerides as 150 mg/dL; Cholesterol HDL <40 mg/dL and blood pressure as a resting systolic 130 mm Hg or a resting diastolic 85 mm Hg, which corresponds to a mean arterial blood pressure (MABP) of 100 mm Hg. For each MetSyn component, the clinical cutoff value was subtracted from the individual’s value and then divided by the population’s standard deviation (SD). The standardized score for each MetSyn component was then averaged to derive a clustered CVD risk score relative to IDF MetSyn definition (CVD risk score). To compile the CVD risk score with units of SD, positively skewed variables (FG and TG) were previously transformed (natural log).

Physical activity: PA levels were assessed at baseline, T1 (16 weeks) and T2 (24 weeks), to determine changes in the following: percentage of participants meeting the PA recommendation, minutes of moderate and vigorous PA performed per week, and minutes per day of sedentary behavior. PA was measured by self-report (indirect methods) and accelerometry (direct methods). Self-reported (subjective) through the short version of the International Physical Activity Questionnaire (IPAQ) (Craig et al., 2003), and objectively by ActiGraph GT3X accelerometers (ActiGraph LLC, Ft. Walton Beach, FL, United States), this device has an internal time clock and extended memory, and it is able to record and store the magnitude of acceleration and deceleration of movements. The recorded data is scored as a “count,” which can be summed in a specific time interval called an “epoch.” In this study, an epoch is equal to 60 s. Participants were instructed to wear the device for 7 days during all waking hours, removing it only during swimming, bathing, or having other contact with water. The accelerometers were mounted on elastic belts and placed at the right hip. For this study wear time was valid if the patient wore the device for 5 days in 1 week (including at least 1 weekend day) to accumulate at least 600 min daily.

Each patient’s accelerometer record was analyzed in terms of time (in minutes) to perform PA of moderate to vigorous intensity per day and then calculate the average time spent (in minutes) of PA during the week. Using this data, patients were categorized as complying or not complying with PA recommendation, 6 (150 min of PA at moderate to vigorous intensity a week) at baseline, T1 and T2, in both IG and CG. Additionally, PA measures were complemented with a PA log that participants completed every week.

### Other Variables

Socio-demographic variables included age, age group, gender, marital status, and main activity.

### Confounding or Modifier Effects Variables

We defined as confounding and modifier variables the following: met PA recommendation measured by both self-reported and objectively with the accelerometer, weight, and BMI at baseline, age, gender, education, main activity.

### Intervention Group: Exercise Referral Scheme

The intervention was based on the assumptions of the Transtheoretical Model and Social Cognitive Theory (Bandura, 1977; Bandura and National Inst of Mental Health, 1986). The information about the procedure of implementation and development of the PA program has already been described and is summarized below.

During the first session, a functional capacity assessment and PA prescription were carried out. The program of 16 weeks had 48 sessions of 60 min, a frequency of three times per week, and an average intensity of moderate to vigorous. Three levels or stages during the 16 weeks of the PA program were developed: Level 1, or Induction; Level 2, or Development; and Level 3, or Maintenance. In addition, each session consisted of three phases of exercise: warm-up and flexibility phase; aerobics phase, and recovery phase. The ER program had 48 sessions of planned PA. We defined a high level of adherence (>50%) to those patients who attended at least half of the sessions (24 sessions or more), while a low level of adherence (<50%) was determined for patients who attended less than 24 sessions of the PA program.

At the end of the 16 weeks of the program, PA specialists encouraged the participants to continue with PA practice at the CSS facilities (at no cost for the following 6 months) or practice regular PA during leisure time in their preferred facilities, but without financial support from IMSS.

### Control Group: Brief Counseling

Participants belonging to PHCUs were randomized to the control group after inclusion criteria were confirmed. Additionally, written and verbal information about the health benefits of PA practice, and how to increase PA levels in a safe way was distributed to the participants. All the patients in this group continued receiving their usual care at PHCU facilities but were also informed about the health benefits of PA and a balanced diet, and how to safely increase PA levels during free time, their participation in this PA program did not include planned PA sessions, therefore adherence to PA program was not estimated. In addition, they were invited to undergo two subsequent measurements at the PHCU facilities, at 16 weeks and 24 to the baseline measurement. Monthly contact with participants was carried out during their consultation at PHCUs.

### Statistical Analyses

Analyses were carried out according to an intention to treat analysis.

A baseline comparability analysis was carried out among the ER and BC, in relation to the variables studied during the baseline, 16 and 14 weeks. To compare means, a *T*-test or analysis of variance (ANOVA) was conducted, while U of Mann Whitney was used to compare variables with not normal distribution.

Due randomization took place in PHCUs and multilevel analyses were used. Longitudinal multilevel analyses were developed to account for the three measurements between intervention and control groups, thus the three follow-up measurements of the outcome measure concerned were defined as dependent variables. The multilevel model allowed taking into account the clustering of individuals within PHCUs through the next levels: (1) the individual and (2) PHCUs. These models were linear for all outcome variables including blood pressure, biochemical and anthropometric measurements, depressive symptoms, the physical and mental component of quality of life, and metabolic syndrome risk score. The Wald statistic was used to determine the statistical significance of intervention effects and level of compliance on the outcome variables.

For the variables, the models were adjusted for confounding or modifier effects variables following the next steps: (1) crude model, adjusted by group (intervention/control), stage (time), and baseline value of the outcome measure concerned, the latest to avoid a regression bias to the estimated mean (Barnett et al., 2005); (2) Additionally adjusted by age, gender, education, main activity, and meet PA recommendation measured both self-reported and objective with an accelerometer. (3) Additionally, adjusted by weight, BMI, and waist circumference. (a) Weight additionally adjusted by BMI and waist circumference; (b) waist circumference additionally adjusted by weight and BMI.

### Ethical Issues

The National Research Commission and Ethics Committee at Mexican Social Security Institution and National Institute of Public Health in Mexico, approved this study and all its procedures, as stated before (Gallegos-Carrillo et al., 2014).

## Results

The characteristics of the study population according to meeting PA recommendations at 16 (T1) and 24 (T2) weeks of follow-up in the BC and the ER groups and by the level of adherence for the ER are shown in Table 1. The proportion of participants in the ER and BC group who met PA recommendations in both T1 and T2 was higher in the ER group (32.4%) than in the BC (25.4%), Body Mass Index (BMI) values were lower in participants meeting PA recommendations in T1 and T2 in comparison with those meeting PA recommendations in T1 only, BMI = 32.6 vs. 28.2 in the ER and BMI = 28.5 vs. 28.2 for the BC group. While differences in glucose levels were only observed in the ER group 109 vs. 103 mg/dL (Table 1), and blood pressure level observed a decrease in the BC, systolic 130 vs. 128 mmHg and diastolic blood pressure 79.9 vs. 76.6 mmHg, respectively (Table 1).

**TABLE 1:** Baseline characteristics among exercise-referral and brief counseling groups according to meeting physical recommendations at the end of the interventions.

	Exercise referral (ER) *n* = 117			Brief counseling (BC) *n* = 115		
Variables	No meet PA recommendation both T1 and T2 *n* = 58 (52.2%)	No meet PA recommendation T1 meet in T2 *n* = 17 (15.3%)	Meet PA recommendation both T1 and T2 *n* = 36 (32.4%)	No meet PA recommendation both T1 and T2 *n* = 59 (53.6%)	No meet PA recommendation T1 meet in T2 *n* = 23 (20.9)	Meet PA recommendation both T1 and T2 *n* = 28 (25.4)
**Socio-demographic**						
Age, mean (SD)	50.8 (10.9)	48.7 (11.6)	50.3 (11.2)	52.5 (9.9)	51.5 (10.6)	50.3 (11.2)
**Age group %**						
35–49	23 (46.9)	8 (16.3)	18 (36.7)	19 (43.2)	10 (22.7)	15 (34.1)
50–65	28 (53.8)	8 (15.4)	16 (30.8)	34 (60.7)	11 (19.6)	11 (19.6)
65 and more	7 (70)	1 (10)	2 (20)	6 (60)	2 (20)	2 (20)
Male (%)	17 (47.2)	5 (13.9)	14 (38.9)	15 (48.4)	8 (25.8)	8 (25.8)
Female (%)	41 (54.7)	12 (16)	22 (29.3)	44 (55.7)	15 (19)	20 (25.3)
**Educational level %**						
Elementary school or less	17 (54.8)	4 (12.9)	10 (32.3)	25 (67.6)	5 (13.5)	7 (18.9)
Middle school	12 (46.1)	3 (11.5)	11 (42.3)	7 (36.8)	4 (21.1)	8 (42.1)
High school/technical	18 (46.1)	7 (17.9)	14 (35.9)	16 (51.6)	7 (22.6)	8 (25.8)
Bachelor or higher	11 (73.3)	3 (20)	1 (6.7)	11 (47.8)	7 (30.4)	5 (21.7)
**Marital status % (C.I. 95%)**						
Single	4 (40)	1 (10)	5 (50)	8 (66.7)	1 (8.3)	3 (25)
Married	45 (51.7)	13 (14.9)	29 (33.3)	40 (51.3)	16 (20.5)	22 (28.2)
Divorced	5 (83.3)	1 (16.7)	0	7 (63.6)	3 (27.3)	1 (9.1)
Widowed	4 (50)	2 (25)	2 (25)	4 (44.4)	3 (33.3)	2 (22.2)
**Main activity % (C.I. 95%)**						
Homemaker	24 (53.3)	6 (13.3)	15 (33.3)	17 (50)	6 (17.6)	11 (32.3)
Student	0	0	1 (100)	1 (100)	0	0
Works outside home	24 (48.9)	10 (20.4)	15 (30.6)	35 (56.4)	13 (20.69)	14 (22.6)
Retired or pensioned	5 (50)	1 (10)	4 (40)	5 (50)	3 (30)	2 (20)
Unemployed	4 (80)	0	1 (20)	1 (33.3)	1 (33.3)	1 (33.3)
Permanently disabled	1 (100)	0	0	0	0	0
**Blood pressure and biochemical measurements mean (SD)**						
Systolic	123.6 (16.7)	126 (27.5)	131 (18.5)	132 (19.3)	130 (15)	128 (17.2)
Diastolic	79 (12.3)	82.1 (14.1)	83.5 (8.6)	77.3 (11.9)	79.9 (12)	76.6 (8.8)
Fasting glucose	108 (35.5)	109 (27.1)	103 (16.8)	106 (23.1)	106.5 (21.6)	106 (28.3)
Cholesterol HDL	47.6 (13.3)	40.6 (8.1)	45.4 (9.3)	46.7 (12.2)	49.7 (12.1)	46.3 (10.9)
Triglycerides	178.9 (71.9)	167.9 (66.1)	162.4 (78.2)	166.5 (62.9)	164.4 (64.4)	151.1 (63.5)
**Anthropometric measures mean (SD)**						
Weight	75.8 (16.3)	82.8 (20.7)	72.9 (13.5)	73.2 (14.3)	72.4 (16.1)	71.5 (9.4)
Body Mass Index	30.2 (5.4)	32.6 (5.8)	28.2 (4.32)	29.9 (4.9)	28.5 (4.9)	28.2 (3.4)
Waist circumference	101.5 (12.3)	105.1 (14.4)	96.2 (11.6)	98.9 (10)	96.3 (9.6)	93.3 (8.2)
**Quality of life and depressive symptoms**						
Physical component summary	48.2 (5.3)	45.7 (6)	47.8 (5.7)	46.8 (4.2)	45.8 (5.5)	47.9 (3.66)
Mental component summary	30.5 (5.2)	33.1 (5.4)	31.2 (5.4)	31.1 (5.4)	31.4 (4.5)	30.6 (3.98)
Depressive symptoms (CES-D 20)	18.8 (7.9)	17 (19.1)	15.4 (6.9)	20.1 (7.9)	21.4 (9.5)	22.8 (10.4)
Diabetes self-reported %	9 (56.2)	1 (6.25)	6 (37.5)	11 (61.1)	3 (16.7)	4 (22.2)

*Physical activity recommendations measured by accelerometers.*

### Longitudinal Analysis in Biological, Biochemical, and Anthropometric Variables

The percentage change observed in systolic and diastolic blood pressure was not statistically significantly different in longitudinal multilevel analyses, although blood pressure levels decreased in the BC group and remained at the same levels in the ER group. We also observed a statistically significant difference in weight and in metabolic syndrome risk score, whose values in participants in the ER group decreased among basal, T1 (16 weeks), and T2 (24 weeks) of follow-up, however, difference by level of compliance was not observed in biological (systolic and diastolic blood pressure, levels of cholesterol HDL AND LDL, biochemical and anthropometrics) variables (Table 2).

**TABLE 2:** Longitudinal changes and multilevel analyses in biological, biochemical and anthropometrics variables among intervention and control groups and by level of compliance.

Variables	Exercise referral	Brief counseling	Crude model^a^	Level of compliance (50% and more)		Crude model (comp.<50% vs. ≥50%)^a^
			*P* value	<50 (*n* = 45)	≥50 (*n* = 72)	*P* value
**Systolic Blood pressure, mm Hg Mean (SD)**						
0 weeks (T0)	124.9 (22)	130.1 (17.9)	0.105	121.1 (24.8)	127.4 (19.9)	0.386
16 weeks (T1)	124.7 (19)	124.6 (18.4)		121.5 (21.2)	126.7 (17.2)	
24 weeks (T2)	124.4 (19.1)	125.9 (18.1)		121.1 (20.6)	126.5 (17.8)	
**Diastolic Blood pressure, mm Hg Mean (SD)**						
0 weeks (T0)	79.9 (13.8)	77.5 (11.2)	0.417	77.4 (17)	81.5 (11.1)	0.905
16 weeks (T1)	76.7 (12.3)	74.2 (12.6)		76.5 (14.6)	76.9 (10.8)	
24 weeks (T2)	76.5 (12.2)	72.1 (12.6)		75.5 (14.3)	77.2 (10.7)	
**Cholesterol HDL (mg/dL) Mean (SD)**						
0 weeks (T0)	45.8 (12.1)	47.1 (11.6)	0.990	45.4 (14.1)	46.1 (10.7)	0.475
16 weeks (T1)	46 (11.1)	47.5 (10.4)		46.4 (12.4)	45.8 (10.2)	
24 weeks (T2)	46.2 (11)	47.4 (10.5)		46.4 (12.2)	46 (10.3)	
**Fasting glucose (mg/dL) Mean (SD)**						
0 weeks (T0)	105.4 (31.3)	106.7 (24.2)	0.243	99.1 (23.4)	109.3 (34.8)	0.299
16 weeks (T1)	105.5 (30.3)	108.2 (28.9)		101.6 (24.9)	107.9 (33)	
24 weeks (T2)	104.6 (31.3)	107.3 (29.8)		98.4 (26.7)	108.5 (33.3)	
**Triglycerides (mg/dL) Mean (SD)**						
0 weeks (T0)	168 (73.3)	161.9 (61.8)	0.016	169.2 (72.7)	167.3 (74.1)	0.829
16 weeks (T1)	179.4 (88.3)	176.6 (82.8)		186.3 (90.2)	170.5 (77.4)	
24 weeks (T2)	178.5 (88)	176.4 (82.5)		182.6 (91.4)	172.6 (76.4)	
**Weight** (Kgs) Mean (SD)						
0 weeks (T0)	76.2 (16.2)	72.6 (13.3)	0.000	81.5 (20.5)	72.9 (11.7)	0.590
16 weeks (T1)	74.8 (15.9)	72.1 (13.1)		79.9 (19.9)	71.7 (11.7)	
24 weeks (T2)	74.9 (15.7)	72.2 (13.2)		79.9 (19.6)	71.8 (11.8)	
**BMI Mean (SD)**						
0 weeks (T0)	30.1 (5.2)	29.2 (4.6)	0.031	31.3 (6)	29.3 (4.5)	0.846
16 weeks (T1)	29.7 (5.2)	29.1 (4.7)		30.9 (6.1)	28.9 (4.4)	
24 weeks (T2)	29.6 (5.1)	29.1 (4.6)		30.7 (5.9)	28.8 (4.4)	
**Waist circumference (cms) Mean (SD)**						
0 weeks (T0)	100.5 (12.5)	97 (9.6)	0.269	103.9 (13.8)	98.3 (11.2)	0.721
16 weeks (T1)	98.1 (11.7)	95.3 (9.4)		101.4 (12.9)	96.1 (10.4)	
24 weeks (T2)	98.2 (11.5)	95.3 (9.4)		100.9 (12.8)	96.5 (10.3)	
**Rate waist/hip Mean (SD)**						
0 weeks (T0)	0.93 (0.08)	0.91 (0.09)	0.759	0.94 (0.08)	0.92 (0.08)	0.530
16 weeks (T1)	0.92 (0.07)	0.90 (0.06)		0.93 (0.07)	0.91 (0.08)	
24 weeks (T2)	0.91 (0.07)	0.91 (0.06)		0.92 (0.08)	0.91 (0.07)	
**Metabolic syndrome Risk Score Mean (SD)**						
0 weeks (T0)	97.5 (17.7)	94.6 (14.6)	0.002	96.1 (17.7)	97.6 (17.9)	0.726
16 weeks (T1)	98.1 (18.7)	99.4 (21.9)		97.1 (44.8)	98.7 (17.5)	
24 weeks (T2)	94.5 (16.5)	99.6 (20.7)		93.3 (16.4)	95.2 (16)	

*Intention to treat analysis. ^a^Adjusted for group, stage (time), and baseline value of outcome measure.*

Longitudinal changes in depressive symptoms and quality of life showed differences statistically significant among Baseline, T1, and T2 in depressive symptoms among the ER and BC groups, and by level of compliance >50% of the ER group in the mean of the mental health component of quality of life (Table 3).

**TABLE 3:** Longitudinal changes and multilevel analyses in quality of life and depressive symptoms variables among exercise referral and brief counseling groups and by level of compliance.

Variables	Exercise referral	Brief counseling	Crude model^a^	Level of compliance (50% and more)		Crude model (comparing <50% vs. ≥50%)^a^
			*P* value	<50 (*n* = 45)	≥50 (*n* = 72)	*P* value
**Physical Component Summary Mean (SD)**						
0 weeks (T0)	46.7 (8.3)	47 (4.6)	0.125	46.9 (5.8)	46.5 (5.2)	0.108
16 weeks (T1)	46.6 (5.2)	45.7 (4.4)		47.5 (5.5)	46.1 (4.9)	
24 weeks (T2)	46.8 (4.9)	45.7 (4.4)		47.6 (5.1)	46.4 (4.8)	
**Mental Component Summary Mean (SD)**						
0 weeks (T0)	30.7 (6.5)	30.8 (4.9)	0.215	28.1 (8.2)	32.4 (4.6)	0.001
16 weeks (T1)	30.9 (5.1)	31.1 (4.4)		30.2 (5.2)	31.3 (4.9)	
24 weeks (T2)	31.1 (5.0)	31.2 (4.4)		30.8 (5.2)	31.3 (4.9)	
**Depressive symptoms (CESD-20) Mean (SD)**						
0 weeks (T0)	18 (7.6)	21.3 (9.3)	0.037	19.3 (9.5)	17.1 (5.9)	0.686
16 weeks (T1)	15.6 (6.7)	17.5 (7.8)		16.4 (7.6)	15.1 (6.1)	
24 weeks (T2)	15.6 (6.9)	17.4 (7.9)		17 (7.8)	14.8 (6.2)	

*Intention to treat analysis. ^a^Adjusted for group, stage (time), and baseline value of outcome measure.*

Multilevel longitudinal analyses according to intention to treat analysis showed statistically significant changes in the biochemical variables measured in the study among ER and BC groups: triglycerides β adjusted = −14.1 (95% C.I. −25.2, −3.1) and Metabolic syndrome risk score β adjusted = −3.5 (95% C.I. −5.7, −1.4), (Table 4). Anthropometric variables as weight β adjusted −1.0 (95% C.I.−1.56, −0.43), β adjusted BMI −0.28 (95% C.I. −0.54, −0.02) and rate waist/hip β adjusted −13.9 (95% C.I. −20.7, −8.8) and perception of depressive symptoms shown difference statistically significant after adjusting for the variables specified in Table 4, including socio-demographic and health status variables, *p*-value < 0.05.

**TABLE 4:** Results of longitudinal multilevel analysis.

Outcomes among intervention and control groups	Crude model^a^ β or OR (95% CI)	*P* value	Adjusted model^b^ β or OR (95% CI)	*P* value	Adjusted model^c^ β or OR (95% CI)	*P* value
Triglycerides (mg/dL)	−13.5 (−24.6, −2)	0.016	−14.1 (−25.2 −3.1)	0.012	−11.8 (−25.6, 2.02)	0.094
BMI	−0.27 (−0.52, 0.2)	0.031	−0.29 (−0.52, −0.05)	0.018	0.28 (−0.54, −0.02)	0.034
Weight	−0.76 (−1.16, −0.36)	0.000	−0.79 (−1.18, 0.39)	0.000	−1.0 (−1.56, −0.43)	0.001
Depressive symptoms (CESD-20)	−3.26 (−5.5. −0.57)	0.019	−3.17 (−5.9, −0.43)	0.025	−2.28 (−7.18, 2.6)	0.305
Rate waist/hip	0.01 (0.0, 0.02)	0.031	0.01 (0.0, 0.02)	0.045	0.007 (−0.00, 0.01)	0.200
Metabolic syndrome risk score	−3.4 (−5.5, −1.2)	0.002	−3.5 (−5.7, −1.4)	0.001	−1.8 (−4.2, 0.39)	0.105
**Outcomes by level of compliance (50%)**						
Mental Component Summary Mean (SD)	−2.0 (−3.2, −0.81)	0.001	−0.6 (−1.57, 0.35)	0.154	−0.56 (−5.1, 4.01)	0.558
Diastolic Blood pressure mm Hg	−2.56 (−4.7, −0.41)	0.019	−1.9 (−4.1, 0.36)	0.100	−1.35 (−3.6, 0.9)	0.246
Waist circumference (cms.)	−1.8 (-3.5, −0.01)	0.048	−1.4 (−3.2, 0.43)	0.136	−0.41 (−2.2, 1.4)	0.650
Rate waist/hip	−0.02 (−0.03 −0.005)	0.006	−0.01 (−0.3, −0.001)	0.029	−0.01 (−0.03, 0.006)	0.190
Depressive symptoms (CESD-20)	−1.66 (−3, −0.28)	0.018	1–56 (−3.2, 0.09)	0.064	−0.88 (-2.4, 0.66)	0.263

*^a^Adjusted for group, stage (time), and baseline value of outcome measure.*

*^b^Additionally adjusted by age, gender, education, employment, acute morbidity and meet physical activity recommendation measured both self-reported and objective with accelerometer.*

*^c^Additionally adjusted by age, gender, education, employment, acute morbidity, meet physical activity recommendation measured both self-reported and objective with accelerometer, weight, BMI and waist circumference (1) Weight additionally adjusted by BMI and waist circumference; (2) waist circumference additionally adjusted by weight and BMI.*

*P value of the interaction term (assessment time × intervention group) and the interaction between (assessment time × adherence).*

The results of longitudinal multilevel analysis by the level of compliance in the ER group observed differences statically significant in diastolic blood pressure β adjusted −2.56 mmHg (95% C.I. −0.47, −0.041), waist circumference β adjusted −1.8 (95% C.I. −3.5, −0.01), depressive symptoms β adjusted −1.66 (95% C.I. −3, −0.28) and the score of the Mental Health Component (MCS) of quality of life, *p* < 0.05, after adjusting for group, stage (time), and baseline value of outcome measure (Table 4).

## Discussion

The results of this study show firstly the feasibility of carrying out an exercise-referral scheme intervention in the context of a social security institution in an upper-middle-income country like Mexico. We observed that both PA programs achieved changes in clinical indicators as triglycerides and anthropometric values Body Mass Index and Metabolic syndrome risk score. While higher levels of adherence of patients who got the ER scheme to increase PA levels accomplished better outcomes in the Mental Health Component in comparison with hypertensive patients who had a lower level of adherence to the ER scheme. In comparison to the BC group, the ER and higher levels of adherence achieved both changes in the perception of social functioning.

Backgrounds in a similar context are lacking, and thus the findings of this cluster-randomized trial would be helpful to encourage the design of programs and strategies to increase PA among patients with chronic diseases in a context such as Mexico.

Physical activity promotion through an exercise-referral scheme, compared to the control group with BC to encourage PA, showed findings which are according to previous studies of effectiveness in which the BC has achieved increased the PA levels compared to the exercise-referral scheme (Galaviz et al., 2013); or as has been found in PA promotion studies, in which increased in PA was reported for both the ER and the BC groups (Hillsdon et al., 2005).

The differences observed in clinical variables such as blood pressure levels, triglycerides, and fasting glucose among those who reported higher levels of adherence to the intervention could be supported by findings in previous studies with similar approaches in which a “Hawthorne” effect could have been present. In fact, the findings of improved clinical and anthropometric factors have been reported in studies carried out mostly in high-income countries (Dugdill et al., 2005).

In a way consistent with the evidence generated in studies of exercise-referral schemes, the ability of the intervention to increase physical health outcomes was low (Buckley et al., 2020). The eligibility assessment carried out in PHCU, might have affected the rate of participation, because 78.6% of patients attended the initial session, in which the exercise was prescribed, and the individualized program was developed. In addition, eligibility criteria as being a hypertensive patient with blood pressure values under control parameters, and having reported a stage of change with a willingness to PA practice would influence in rates of participation in ER programs, which has not been applied for participants in the BC group.

Likewise, in the follow-up, the rates of participation among the ER and BC were different, while the control group observed a follow-up rate at 24 weeks of 90%, the intervention group saw a decrease to 62%. Independent of changes of behavior and contrary to expectations, a behavioral theory-based intervention carried out with an exercise-referral scheme had less impact hooking the subjects as participants in the study. Studies in primary health care settings have suggested that motivational activities including the provision of exercise are more effective than interventions with only exercise aims (Lee et al., 2012). However, in this cluster-randomized trial provision of BC and health promotion materials combined with monthly monitoring attained a higher rate of participation than closer supervision and prescription. Therefore, as was shown in this study, unless a certain level of adherence to an exercise-referral scheme can be guaranteed; BC would be a good alternative to promote PA and achieve the recommended levels (Pate et al., 1995).

The modest effect of an exercise-referral scheme using a behavioral theory-based approach to achieve a recommended level, as stated before in National Exercise-Referral Schemes depends on the level of adherence; one of the most questionable issues of exercise-referral schemes. In this cluster trial, we set a goal of 50% of compliance (attendance of at least 24 sessions) which resulted in 78.2% of participants who began the exercise program achieving this level of adherence; a top-level compared to the levels observed in other trials. In Taylor et al. (1998) observed a 28% of adherence and 33% Lamb et al. (2002). However, the changes observed in the intervention group along time in reducing high blood pressure along the follow-up period keep no consistency whether among ER and BC, nor by level of adherence or compliance; therefore a larger sample size that allows a longitudinal stratified analysis and determination of the factors associated with adherence to these kinds of initiatives meant to increase physical health outcomes, i.e., blood pressure and fasting glucose of hypertensive populations, is necessary.

### Strengths and Limitations

This cluster-randomized trial has several strengths. To our knowledge, this is perhaps the first study to carry out an intervention to increase PA through an exercise-referral scheme among sedentary patients with a recent diagnosis of hypertension; in an upper-middle-income country.

This study used a control group with the same measurements throughout the study, which allowed for the assessment of the intervention effects from several dimensions during the three stages of the study. Particularly it had the strength of assessing PA levels through an objective method, thereby reducing the bias of self-reported PA measures.

It measured the sustainability effects and the level of adherence to the intervention, reinforcing the capacity of measuring the effects carefully. The trial was clustered by practice to reduce the risk of the intervention being contaminated; however, this mechanism of allocation was not enough to avoid changes in lifestyles in the control group, thus changes in outcome variables were observed in the BC group, which as it has been mentioned received a less intensive PA program under a BC approach, influencing not only in PA variables but in other lifestyles, findings that have been previously reported (Gallegos-Carrillo et al., 2017). Finally, and according to the protocol study, the intervention was extended to the control group, after the follow-up of 24 weeks ended.

## Conclusion

This intervention achieved changes among the hypertensive patients who participated in the exercise referral program in comparison with those in the BC group. Changes observed among PA programs at time 0, 16, and 24 weeks pointed out a better performance of the ER in comparison to the BC program in clinical indicators and anthropometric variables, such as triglycerides and the Metabolic syndrome risk score, BMI, and also depression symptoms. About the objective aimed to determine differences in the outcome variables according to level of adherence to the exercise referral scheme, we observed that whether in clinical outcomes as blood pressure levels, waist circumference, and the rate waist/hip and in depressive symptoms and health-related quality of life perception of hypertensive patients who achieved at least 50% adherence to the ER have a better outcome than those with a lower level of adherence. However, although these results did not keep statistical significance in the adjusted models, they at least allow us to glimpse the benefits of PA when programs achieved higher attachment, which could be an important finding to promote a higher engagement in PA programs in the population with chronic conditions.

## Data Availability Statement

The original contributions presented in the study are included in the article/Supplementary Material, further inquiries can be directed to the corresponding author.

## Ethics Statement

The studies involving human participants were reviewed and approved by The National Research Commission and Ethics Committee at Mexican Social Security Institution, and National Institute of Public Health in Mexico, approved this study and all its procedures. The patients/participants provided their written informed consent to participate in this study.

## Author Contributions

KG-C wrote the original protocol and manuscript, collected the data, designed statistical models, and performed analyses. CG-P contributed to the original protocol and wrote the first and final draft. NS-D-S wrote the first draft with contributions from KG-C. JS contributed to the original protocol and wrote the first draft with contributions from KG-C. FL contributed to the original protocol, supported in developing the strategy to analyze the data, and wrote the first draft with contributions from KG-C. All authors contributed to the article and approved the submitted version.

## Conflict of Interest

The authors declare that the research was conducted in the absence of any commercial or financial relationships that could be construed as a potential conflict of interest.

## Publisher’s Note

All claims expressed in this article are solely those of the authors and do not necessarily represent those of their affiliated organizations, or those of the publisher, the editors and the reviewers. Any product that may be evaluated in this article, or claim that may be made by its manufacturer, is not guaranteed or endorsed by the publisher.
