# Supplementary material for: Levels of Adherence of an Exercise Referral Scheme in Primary Health Care: Effects on Clinical and Anthropometric Variables and Depressive Symptoms of Hypertensive Patients
Source: Front Physiol. 2021 Dec 21;12:712135. doi: 10.3389/fphys.2021.712135 (PMC8724582; doi:10.3389/fphys.2021.712135)
Supplement: Supplementary file 1 [file Data_Sheet_1.pdf]

Table 3B. Longitudinal changes and multilevel analyses in quality of life and depressive symptoms variables among exercise referral and brief counseling groups and by level of compliance. Intention to treat analysis.

**Variables**	**Exercise referral**	**Brief counseling**	**Crude model** [a]	**Level of compliance** (50% and more)		**Crude model (comparing <50% vs ≥ 50%)** [a]
			**P value**	**<50 (**n=45)	**≥ 50** (n= 72)	**P value**
**Physical Component Summary** Mean (SD)						
0 weeks (T0)	46.7 (8.3)	47 (4.6)		46.9 (5.8)	46.5 (5.2)	
16 weeks (T1)	46.6 (5.2)	45.7 (4.4)	0.125	47.5 (5.5)	46.1 (4.9)	0.108
24 weeks (T2)	46.8 (4.9)	45.7 (4.4)		47.6 (5.1)	46.4 (4.8)	
**Mental Component Summary** Mean (SD)						
0 weeks (T0)	30.7 (6.5)	30.8 (4.9)		28.1 (8.2)	32.4 (4.6)	
16 weeks (T1)	30.9 (5.1)	31.1 (4.4)	0.215	30.2 (5.2)	31.3 (4.9)	0.001
24 weeks (T2)	31.1 (5.0)	31.2 (4.4)		30.8 (5.2)	31.3 (4.9)	
**Vitality** Mean (SD)						
0 weeks (T0)	31.7 (24.5)	22.8 (23.0)		30.1 (22.4)	32.6 (25.6)	
16 weeks (T1)	23.2 (25.6)	13.9 (20.2)	0.001	26.7 (28.4)	22.4 (24.9)	0.928
24 weeks (T2)	23.2 (23.5)	16.5 (23.8)		22.9 (19.2)	23.3 (24.4)	
**Social funcioning** Mean (SD)						
0 weeks (T0)	18.7 (24.2)	16.1 (25.4)		18.8 (23.4)	18.7 (24.8)	
16 weeks (T1)	12.2 (20.4)	8.1 (21.4)	0.000	11.7 (18.2)	12.3 (20.9)	0.000
24 weeks (T2)	11.9 (21.4)	4.5 (13.4)		22.9 (26.3)	9.7 (19.6)	
**Role Emotional** Mean (SD)						
0 weeks (T0)	18.8 (9.5)	19.2 (9.5)		17.3 (9.7)	19.7 (9.3)	
16 weeks (T1)	19.7 (9.0)	21.4 (7.5)	0.003	20.8 (7.5)	19.4 (9.3)	0.089
24 weeks (T2)	19.4 (10.5)	21.6 (6.9)		16.6 (11.9)	19.9 (10.1)	
**Mental health** (SD)						
0 weeks (T0)	76.9 (22.0)	80.7 (22.4)		71.8 (24.1)	79.9 (20.1)	0.860
16 weeks (T1)	82.3 (20.6)	88.2 (18.7)	0.025	75 (21.2)	84 (20.1)	
24 weeks (T2)	79.7 (20.4)	90.2 (16.9)		80.2 (20.3)	79.5 (20.5)	
**Depressive symptoms (CESD-20)** Mean (SD)						
0 weeks (T0)	18 (7.6)	21.3 (9.3)		19.3 (9.5)	17.1 (5.9)	0.686
16 weeks (T1)	15.6 (6.7)	17.5 (7.8)	0.037	16.4 (7.6)	15.1 (6.1)	
24 weeks (T2)	15.6 (6.9)	17.4 (7.9)		17 (7.8)	14.8 (6.2)	
